# Assessing Causality Between Second-Hand Smoking and Potentially Associated Diseases in Multiple Systems: A Two-Sample Mendelian Randomization Study

**DOI:** 10.1093/ntr/ntad193

**Published:** 2023-10-03

**Authors:** Shilin Wang, Peiwen Yang, Hao Liu, Zhiwen Wang, Poyi Hu, Ping Ye, Jiahong Xia, Shu Chen

**Affiliations:** Department of Cardiovascular Surgery, Union Hospital, Tongji Medical College, Huazhong University of Science and Technology, Wuhan, People’s Republic of China; Department of Cardiovascular Surgery, Union Hospital, Tongji Medical College, Huazhong University of Science and Technology, Wuhan, People’s Republic of China; Department of Cardiovascular Surgery, Union Hospital, Tongji Medical College, Huazhong University of Science and Technology, Wuhan, People’s Republic of China; Department of Cardiovascular Surgery, Union Hospital, Tongji Medical College, Huazhong University of Science and Technology, Wuhan, People’s Republic of China; Department of Cardiovascular Surgery, Union Hospital, Tongji Medical College, Huazhong University of Science and Technology, Wuhan, People’s Republic of China; Department of Cardiology, The Central Hospital of Wuhan, Tongji Medical College, Huazhong University of Science and Technology, Wuhan, People’s Republic of China; Department of Cardiovascular Surgery, Union Hospital, Tongji Medical College, Huazhong University of Science and Technology, Wuhan, People’s Republic of China; Department of Cardiovascular Surgery, Union Hospital, Tongji Medical College, Huazhong University of Science and Technology, Wuhan, People’s Republic of China

## Abstract

**Introduction:**

The global disease burden may be exacerbated by exposure to passive smoking (SHS), with the workplace being a primary location for such exposure. Numerous epidemiological studies have identified SHS as a risk factor for diseases affecting various systems, including cardiovascular, respiratory, immune, endocrine, and nervous systems. The conventional observational study has certain methodological constraints that can be circumvented through a Mendelian randomization (MR) study. Our MR study intends to investigate the causal link between workplace exposure to SHS and the potential associated diseases.

**Aim and Methods:**

Summary statistics data involving European participants were sourced from three databases: the UK Biobank, the FinnGen study, and the European Bioinformatics Institute. Genetic variants linked with exposure to SHS in the workplace were identified as instrumental variables. The MR was carried out using inverse variance weighted (IVW), MR-Egger, and weighted median methods. Sensitivity tests were also undertaken within the MR to evaluate the validity of the causality.

**Results:**

According to the IVW model, genetically determined atrial fibrillation (AF) and stroke (*p* = 6.64E−04 and 5.68E−07, odds ratio = 2.030 and 2.494, 95% confidence interval = 1.350 to 3.051 and 1.743 to 3.569) were robustly associated with exposure to SHS in the workplace. Suggestive associations were found between workplace SHS and myocardial infarction (MI), asthma, and depression.

**Conclusions:**

The MR study demonstrates that exposure to SHS in the workplace is a significant risk factor for AF and stroke in European individuals. Whether workplace exposure to SHS influences other diseases and the causality between them requires further exploration.

**Implications:**

This study explored the causality between exposure to SHS in the workplace and potential associated diseases in multiple systems, including MI, AF, stroke, lung cancer, asthma, allergic disease, type 2 diabetes, and depression, using an MR study. The MR study can circumvent the methodological constraints of observational studies and establish a causal relationship. The two-sample MR analysis provides evidence supporting the causal association of frequent workplace SHS with AF and stroke. Individuals exposed to SHS in the workplace may also have a heightened risk of MI, asthma, and depression. However, whether SHS affects other diseases and the causality between them requires further investigation. To our knowledge, this is the first two-sample MR study to determine the causal relationship between SHS and potential diseases. Exposure to SHS in the workplace is a prevalent issue and may contribute to a global disease burden. The reduction of exposure following the introduction of smoke-free laws has led to a decrease in the admission rate for cardiac events and an improvement in health indicators. It is crucial to further advance smoke-free policies and their implementation.

## Introduction

Exposure to second-hand smoke (SHS) is a prevalent issue and may lead to a global disease burden.^1^ In 2015, an evaluation of passive smoke exposure in the workplace among the US population indicated nearly one-fifth of non-smoking employees experienced exposure to SHS at work, with over half of these subjected to it at least twice a week, despite the introduction of comprehensive smoke-free legislation.^2^ It was estimated that passive smoking resulted in hundreds of thousands of fatalities within a single year.^1^ Evidence about health risks due to SHS has been gathered from numerous observational studies. The higher number of estimated fatalities in adults due to exposure to SHS was attributed to coronary heart disease, lung cancer, and asthma.^1,3,4^ Moreover, atrial fibrillation (AF),^5^ stroke,^6^ allergic disease,^7,8^ type 2 diabetes,^9^ and depression^10,11^ have also been reported as outcomes of passive smoking. However, contradictory conclusions have been suggested by other studies.^12,13^

Due to the methodological limitations of observational studies, such as cross-sectional and cohort studies, which do not demonstrate causality and fail to eliminate numerous confounding factors, the Mendelian randomization (MR) method was developed to mitigate the effects of confounding factors and establish a causal relationship. In MR, the causal effects of the exposure on the outcome are evaluated by instrumental variables (IVs). As genetic variants are randomly distributed during meiosis and cannot be altered post-conception or during the progression of the disease, they are typically used as IVs to enhance the reliability of the results. Genetic variants can only serve as IVs when they meet the following conditions^14–16^: First, the variants must be strongly associated with exposure. Second, the variants must not be associated with any confounders of the exposure-outcome association. Last, the variants cannot directly affect the outcome, except potentially through the exposure pathway. Additionally, based on the available public genetic data, MR can be widely applied.

In this two-sample MR analysis, our objective was to examine whether SHS in the workplace might influence the risk of potentially related diseases reported in prior observational studies. These include myocardial infarction (MI), AF, stroke, lung cancer, asthma, allergic disease, type 2 diabetes, and depression.

## Methods

### Study Design Overview

**Figure 1.**
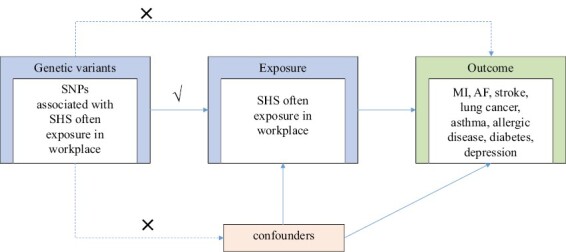
Study design of a two-sample Mendelian randomization study between second-hand smoking (SHS) and eight types of diseases in multiple systems. The “×” signifies that genetic variants are not correlated with confounders or cannot be directly involved in the outcome. The “√” signifies that genetic variants are highly associated with exposure; thus, they can influence outcomes solely through exposure.

The design of the two-sample MR study is shown in Figure 1. In short, the causal effects of SHS in the workplace and MI, AF, stroke, lung cancer, asthma, allergic disease, type 2 diabetes, and depression were estimated, respectively. Genetic variants should strictly adhere to the three assumptions provided above. We used summary statistic data sets from recent genome-wide association studies (GWAS) of SHS in the workplace and the potentially associated diseases.

### Data Sources and SNP Selection

The UK Biobank (UKB) study is a prospective cohort study that gathered and provided genetic and other data on over 500 000 participants residing in the UK.^17^ From the second round of GWASs results from the UKB (http://www.nealelab.is/uk-biobank), we extracted SHS-related independent single nucleotide polymorphisms (SNPs): workplace had a lot of cigarette smoke from other people smoking (Self-reported: often, sex: male and female, Phenotype Code: 22611_2; *n*case = 14 941; *n*control = 74 862), which has been previously utilized in Ang Li’s study.^18^ The sample characteristics are shown in Table 1. We used *p* < 5.0 × 10^−5^ as the benchmark for genome-wide significance to select the variants related to “workplace had a lot of cigarette smoke from other people smoking.” To guarantee a minimum of 10 residual SNPs for examination following the clumping of SNPs for independence and harmonization with outcome data, the threshold was eased from *p* < 5.0 × 10^−8^ to *p* < 5.0 × 10^−5^ to secure an adequate quantity of SNPs. 1077 associated variants were selected. We then conducted the linkage disequilibrium test on these SNPs to clump SNPs for independence (*r*^2^ < 0.001, kb > 10 000). We searched all of the remaining 106 SNPs associated with SHS in the Phenoscanner database (http://www.phenoscanner.medschl.cam.ac.uk/) to identify whether these SNPs associated with confounding factors or directly affected the outcome (*p* < 5 × 10^−8^). No SNPs were removed in this step. The SNPs significantly associated with SHS in the workplace are shown in Supplementary Table S1.

**Table 1.** Brief Description of the Genome-wide Association Study Data Used in this Study

GWAS ID	Year	Trait	Sample size	*n*case	*n*control	Number of SNPs	*R* ^2^	*F*
ukb-d-22611_2	2018	Workplace had a lot of cigarette smoke from other people smoking: Often	89 803	14 941	74 862	13 566 864	—	—
finn-b-I9_MI	2021	Myocardial infarction	200 641	12 801	187 840	16 380 433	9.81E-05	19.68
finn-b-I9_AF	2021	Atrial fibrillation and flutter	138 994	22 068	116 926	16 379 794	1.74E-04	24.24
ebi-a-GCST006906	2018	Stroke	446 696	40 585	406 111	8 211 693	2.30E-05	10.29
finn-b-C3_LUNG_NONSMALL	2021	Non-small cell lung cancer	218 792	1627	217 165	16 380 466	9.62E-05	21.05
finn-b-J10_ASTHMA	2021	Asthma	156 078	20 629	135 449	16 380 176	9.84E-05	15.36
ebi-a-GCST005038	2017	Allergic disease	360 838	180 129	180 709	8 133 670	6.83E-05	24.65
ebi-a-GCST005413	2018	Type 2 diabetes	70 127	12 931	57 196	14 277 791	2.81E-04	19.70
finn-b-F5_DEPRESSIO	2021	Depression	215 644	23 424	192 220	16 380 457	7.41E-05	15.97

The GWAS summary statistics of outcome data in our study encompassed eight phenotypes: MI, AF, stroke, lung cancer, asthma, allergic disease, type 2 diabetes, and depression. We searched for “Myocardial infarction,” “Atrial fibrillation and flutter,” “Stroke,” “Non-small cell lung cancer,” “Asthma,” “Allergic disease,” “Type 2 diabetes,” and “Depression” as the keywords, selecting data that used participants of European descent from the IEU Open GWAS database^19,20^ (https://gwas.mrcieu.ac.uk/). The sample characteristics of exposure and outcome GWAS are shown in Table 1. The SNPs for which proxy SNPs were unavailable in the outcome GWAS were removed, and then the exposure and outcome data were harmonized before evaluating the association between SHS and relative diseases. Simultaneously, palindromic SNPs with intermediate allele frequency were excluded. The comprehensive information on SNPs we ultimately utilized in our study is shown in Supplementary Tables S2–S9.

### MR Analysis and Sensitivity Test

In this study, eight independent two-sample MR analyses were carried out using the “TwoSampleMR” package^19^ with R software (version 4.2.2, RStudio). After the effect of each SNP was calculated using the Wald ratio method, the individual effect of each SNP was used to generate the final beta estimate (beta _outcome_/beta _exposure_) through a meta-analysis utilizing the inverse variance weighted (IVW) method. As the inverse variance weighted-fixed effects (IVW-FE) method is the most efficient and widely applied method, it was used as our primary analytical approach. Because the outcome was binary,^21^ we converted the final beta estimate to the odds ratio (OR). The MR-Egger regression method^22^ and the weighted median (WM) model^21^ were also implemented to further assess the causal relationship between SHS exposure in the workplace and the potentially associated diseases. MR-Egger is a method capable of effectively testing the null causal hypothesis and serves as the foundational method for horizontal pleiotropy in our analysis.^22^ The WM model supplements the assumption of the IVW method that all SNPs are valid IVs, and it provides a consistent effect even if half of the SNPs are pleiotropic.^21^ The threshold of statistical significance of pleiotropy is *p* < .05. Cochran *Q* statistics and *I*^2^ statistics are used to quantify the level of heterogeneity. In the sensitivity test, the “leave-one-out” analysis is utilized to analyze the influence of a single SNP on the results of MR analysis by removing every single SNP at a time using the IVW method. We removed the SNPs highly sensitive through it. Additionally, we used the MR pleiotropy residual sum and outlier (MR-PRESSO) test^23^ to remove the outlier (Nb Distribution = 10 000, Significant Threshold = 0.05), which was conducted by the package “MR-PRESSO.” The statistical threshold for “MR-PRESSO” is *p* < .05. Finally, *F-*statistics were utilized to evaluate the strength of SNPs. We calculated the *F* statistic with the formula:


$$\begin{array}{l} F=\left(N-k-1\right)/N\times R^{2}/\left(1-R^{2}\right) \end{array}$$

(*N* = sample size of the exposure, *k* = the number of selected SNPs, and *R*^2^ represents the phenotype variance induced by the SNPs.) When *R*^2^ is not available, we used the formula:


$$\begin{array}{l} R^{2}=2\times MAF\times \left(1-MAF\right)\times {\left(\beta /SD\right)}^{2} \end{array}$$

(β = the effect value of the genetic variant of the exposure, MAF = the effect allele frequency of selected SNPs, SD=SE×√N, *SE* = the standard error of the genetic variant of the exposure, *N* = sample size of the exposure). The SNPs with *F-*statistics >10 were identified as powerful instruments, and then we included them. Each *R*^2^ and *F* of exposure for different outcomes are shown in Table 1.

Because the outcome was binary, we reported the effect estimates in ORs and 95% confidence intervals (CIs). The main results had robust significance at *p* < .002 (0.05/24) after a Bonferroni correction due to the eight diseases studied and the three statistical models used. The *p* values between 0.002–0.05 were considered suggestively significant, and the *p* value above .05 was considered not significant.

## Results

### Pleiotropy Heterogeneity and Sensitivity Analysis

**Figure 2.**
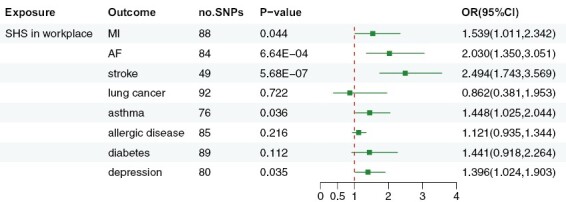
Associations of SHS exposure in the workplace with eight types of diseases in multiple systems. Often exposure to SHS in the workplace was a risk factor for atrial fibrillation and stroke. There are suggestive associations between SHS in the workplace and asthma, myocardial infarction, and depression. However, there may be no association between SHS and lung cancer, allergic diseases, and type 2 diabetes.

After investigating the residual SNPs associated with SHS, we discovered that 88, 84, 49, 92, 76, 85, 89, and 80 SNPs were linked with MI, AF, stroke, lung cancer, asthma, allergic disease, type 2 diabetes, and depression, respectively (Figure 2). These were utilized as IVs for SHS in the workplace. *F*-statistics for SHS’s IVs exceeded the benchmark of 10, signifying that the IVs in all groups were robust instruments, which minimized the bias of the IVs’ estimates.

Both MR-Egger and IVW-FE demonstrated *p* > .05 in Cochran’s *Q* test (Table 2), signifying that all genetic variants of any group did not exhibit significant heterogeneity. The MR-Egger intercept is employed to test the pleiotropy and the outcomes are shown in Table 2.

**Table 2.** Heterogeneity and Pleiotropy Test of SHS Genetic Instrumental Variables in GWAS for Eight Outcome Diseases

Exposure	Outcome	Heterogeneity test						Pleiotropy test		
		MR-Egger			IVW			MR-Egger		
		*Q*	*Q*_*df*	*p* Value	*Q*	*Q*_*df*	*p* Value	Intercept	*SE*	*p* Value
SHS in workplace	MI	92.02	86	.31	92.45	87	.32	−3.58E-03	0.006	.531
	AF	82.72	82	.46	82.73	83	.49	−3.70E-04	0.005	.946
	Stroke	19.91	47	1.00	20.17	48	1.00	−2.53E-03	0.005	.616
	Lung cancer	71.39	90	.93	72.05	91	.93	7.83E-03	0.010	.420
	Asthma	57.69	74	.92	58.02	75	.93	2.62E-03	0.005	.569
	Allergic disease	101.56	83	.08	101.61	84	.09	4.84E-04	0.002	.845
	Diabetes	95.31	87	.25	95.36	88	.28	1.40E-03	0.006	.821
	Depression	70.31	78	.72	70.44	79	.74	1.47E-03	0.004	.714

For the sensitivity analysis, we utilize the “leave-one-out” analysis and “MR-PRESSO.” In the “leave-one-out” test, we identified the sensitive SNPs as their black dot on the opposite side of 0 compared to the result of all IVs. All sensitive SNPs are eliminated until each SNP cannot influence the result independently. A comprehensive analysis incorporating all remaining SNPs is shown in Supplementary Figure 1. In the “MR-PRESSO” test, we eliminated any outliers until there were no outliers in the substantial MR analysis.

As the null causal hypothesis can be efficiently tested by the MR-Egger regression method,^22^ we employed MR-Egger to test the impact of confounding factors on outcomes, the results of which are shown in Supplementary Figure S2. MR-Egger and IVW-FE were also utilized to test the reporting bias (Supplementary Figure S3). In addition, the forest plots are shown in Supplementary Figure S4.

### MR Analysis of SHS in the Workplace and Potential Diseases

We explored the genetic correlation between SHS in the workplace and potential diseases. As shown in Figure 2, according to the IVW-FE model, we discovered that SHS has a robust significant positive causal correlation with AF and stroke (*p* = 6.64E-04 and 5.68E-07, OR = 2.030 and 2.494, 95% CI = 1.350 to 3.051 and 1.743 to 3.569). The genetic correlation is generally stronger between SHS and stroke. Asthma (*p* = .036, OR = 1.448, 95% CI =1.025 to 2.044), MI (*p* = .044, OR = 1.539, 95% CI = 1.011 to 2.342), and depression (*p* = .035, OR = 1.369, 95% CI = 1.024 to 1.903) were suggestive outcomes of SHS exposure. However, SHS in the workplace might not be associated with lung cancer (*p* = .722, OR = 0.862, 95% CI = 0.381 to 1.953), allergic disease (*p* = .216, OR = 1.121, 95% CI = 0.935 to 1.344), and type 2 diabetes (*p* = .112, OR = 1.441, 95% CI = 0.918 to 2.264).

## Discussion

The two-sample MR analysis provides evidence supporting the causal link between often SHS in the workplace and stroke and AF. Individuals exposed to SHS at work may also face a heightened risk of asthma, MI, and depression. To the best of our understanding, this is the first two-sample MR study to pinpoint the causal relationship between SHS and potential diseases. Previous MR studies primarily concentrated on the effect of smoking on smokers’ health,^24^ and the number of passive smokers globally may greatly outnumber smokers,^25^ even with the introduction of smoke-free legislation, the number of workers in certain industries exposed to SHS remains high.^2^ Therefore, the health concerns of passive smokers require greater attention, which is the purpose and original intention of our study. Exposure to SHS in the workplace is a widespread issue and could contribute to a global disease burden. The reduction of exposure following smoke-free laws leads to a decrease in admission rates for cardiac events and an improvement in health indicators. It’s crucial to further promote smoke-free policies and their implementation.

To date, numerous epidemiological studies have determined that SHS is a risk factor for several diseases, including MI,^26^ AF,^5^ stroke,^6^ lung cancer, asthma,^1,3,4^ allergic disease,^7,8^ type 2 diabetes,^9^ and depression.^10,11^ However, some studies have reached different conclusions. A prospective study suggested that passive exposure to tobacco smoke is not linked with stroke.^12^ Allergic rhinitis did not have a strong association with SHS in a cross-sectional study.^13^ The conventional observational study has some methodological limitations, such as being influenced by confounding factors and reverse causality, which can be circumvented by an MR study.

Exposure to SHS may diminish the blood’s capacity to transport oxygen, impair the myocardium’s ability to utilize oxygen,^27^ and decrease heart rate variability,^28^ leading to a heightened risk of ischemic heart disease and cardiac arrhythmia. Exposure to SHS may trigger an abnormal response of immune cells in allergic disease^29,30^ and cancer,^31^ leading to obstructions to pathogen and dead cell clearance.^32,33^ Exposure to cigarette smoke may also activate the oxidative stress of β-cell, impairing their function and increasing the risk of type 2 diabetes.^34^ Nicotine may alter the metabolism of the cerebral cortex associated with depressive behavior.^35^ However, employing three different methods of MR analysis (IVW-FE, MR-Egger, and WM), we found no causality between SHS and lung cancer, allergic disease, and type 2 diabetes. There may be some disparities between in vivo and in vitro experiments and the actual human situation.

This study has several limitations. First, we used *p* < 5 × 10^−5^ as the standard of genome-wide significance to select the variants related to exposure, which made the SNPs less specific. Second, this study only included participants of European descent, and instruments identified in European populations are not necessarily appropriate for non-European populations. Further studies of other descents are needed to verify the causality. Third, we did not stratify the causal association between SHS and potential diseases by gender and subtype, although some studies suggested that may affect the causality. Some studies suggested SHS may have dose-response relationships with specific symptoms,^36^ of which we did not compare in our study. Moreover, because the SNPs identified have a small variance, the effect of most of them is moderate,^37^ which means a more reliable instrument and more samples are needed for more precise results. Furthermore, a minor sample overlap between SHS exposure and associated diseases may result in bias.^38^ Within extensive biobanks with sample sizes surpassing 100 000, most two-sample MR modes can be safely employed for one-sample MR, even with significant confounding, particularly the IVW-FE and WM estimator which are more resistant to pleiotropy than one-sample MR methods.^39^ Considering that the UK Biobank is a vast data set with over 300 000 participants, and a range of suitable methods performing well in one-sample MR applied to our study, we are confident in the reliability of our conclusions. Last, as the exposure in our study, is SHS in the workplace, we only report on diseases in adults. According to other studies, children are more likely to be exposed to SHS, which also results in a significant disease burden.^1^ Some previous MR studies have explored the causal relationship between maternal smoking during pregnancy and the diseases and behaviors of offspring such as autism and smoking initiation.^40,41^ These studies concluded that there is insufficient evidence to support a causal association between them. In addition, several observational studies about the association between childhood SHS exposure and the risk of SHS-related disease in adulthood demonstrated that childhood SHS exposure may increase the risk for allergic disease,^7^ asthma,^42^ adult AF,^43^ and so on. Due to the confounding factors and the unclear causal direction in observational studies, causal inference methods can be used in further studies to expand the evidence base. Allergic disease is more likely to be observed in children according to previous studies.^7^

Nonetheless, this study has some advantages. First, the number of SNPs involved in our study is relatively large. Second, the impact of confounding factors is minimal according to the scatter plot (Supplementary Figure S2). Third, to avoid overlap in the study population, we selected samples from two different databases. Fourth, it is straightforward to obtain abundant genetic data from the public genetic data set using the MR method and the summary statistics are also applicable to individual-level data in terms of statistical power.^44^ Additionally, in MR analysis, the causal direction is clear.

In conclusion, our MR analysis found a causal effect of often exposure to SHS in the workplace on the increased risk of AF and stroke, indicating that SHS can be a health hazard in European individuals. Consistent evidence has shown that smoke-free laws can reduce exposure to SHS in workplaces,^45–48^ leading to a reduction in hospital admissions for cardiac events and an improvement in health indicators.^46^ However, there is still a significant gap between the current state and the full implementation of the smoking ban, which necessitates sensible planning and collective efforts.

## Supplementary Material

A Contributorship Form detailing each author’s specific involvement with this content, as well as any supplementary data, are available online at https://academic.oup.com/ntr.

ntad193_suppl_Supplementary_Figures_S1-S4

ntad193_suppl_Supplementary_Tables_S1-S9

## Data Availability

The data from the UK Biobank can be obtained from http://www.nealelab.is/uk-biobank. The data from the IEU Open GWAS database can be obtained from https://gwas.mrcieu.ac.uk/.
