# Supplementary material for: Assessing Causality Between Second-Hand Smoking and Potentially Associated Diseases in Multiple Systems: A Two-Sample Mendelian Randomization Study
Source: Nicotine Tob Res. 2023 Oct 3;26(6):678–84. doi: 10.1093/ntr/ntad193 (PMC11109494; doi:10.1093/ntr/ntad193)
Supplement: ntad193_suppl_Supplementary_Figures_S1-S4 [file ntad193_suppl_supplementary_figures_s1-s4.pdf]

Supplementary Figures

Figure S1: Single plot of causal effects of SHS associated SNPs on eight diseases. 1
Figure S2: Scatter plot of causal effects of SHS associated SNPs on eight diseases. 2
Figure S3: Funnel plot of causal effects of SHS associated SNPs. 3
Figure S4: Forest plots for association of SHS with potential related diseases................................. 4

Supplementary Figure S1

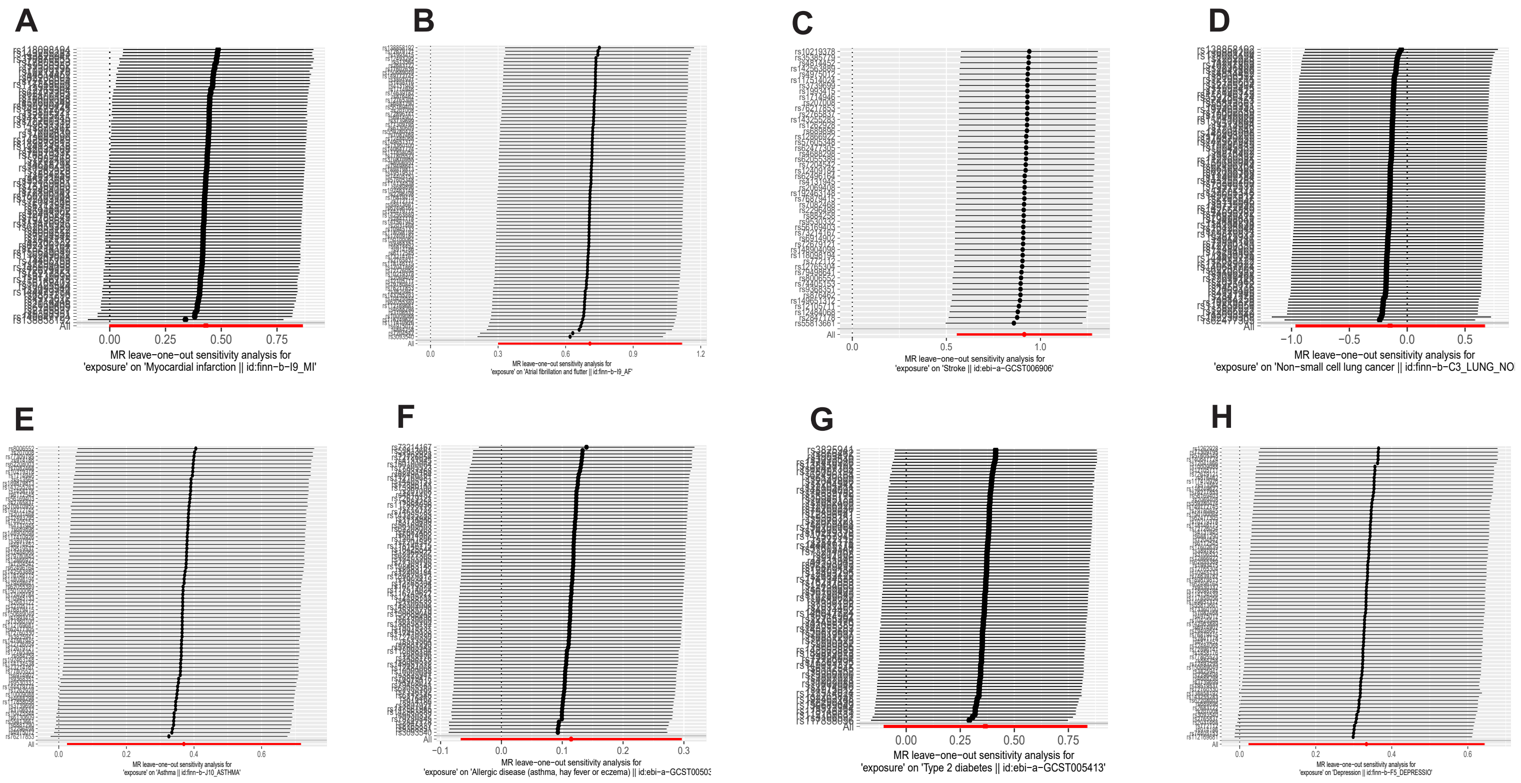

Figure S1. Single plot of causal effects of SHS associated SNPs on eight diseases. Figure showed the leave-one-out sensitivity analysis for SHS on (A) MI, (B) AF, (C) stroke, (D) non- small cell lung cancer, (E) asthma, (F) allergic disease, (G) type 2 diabetes and (H) depression.

Supplementary Figure S2

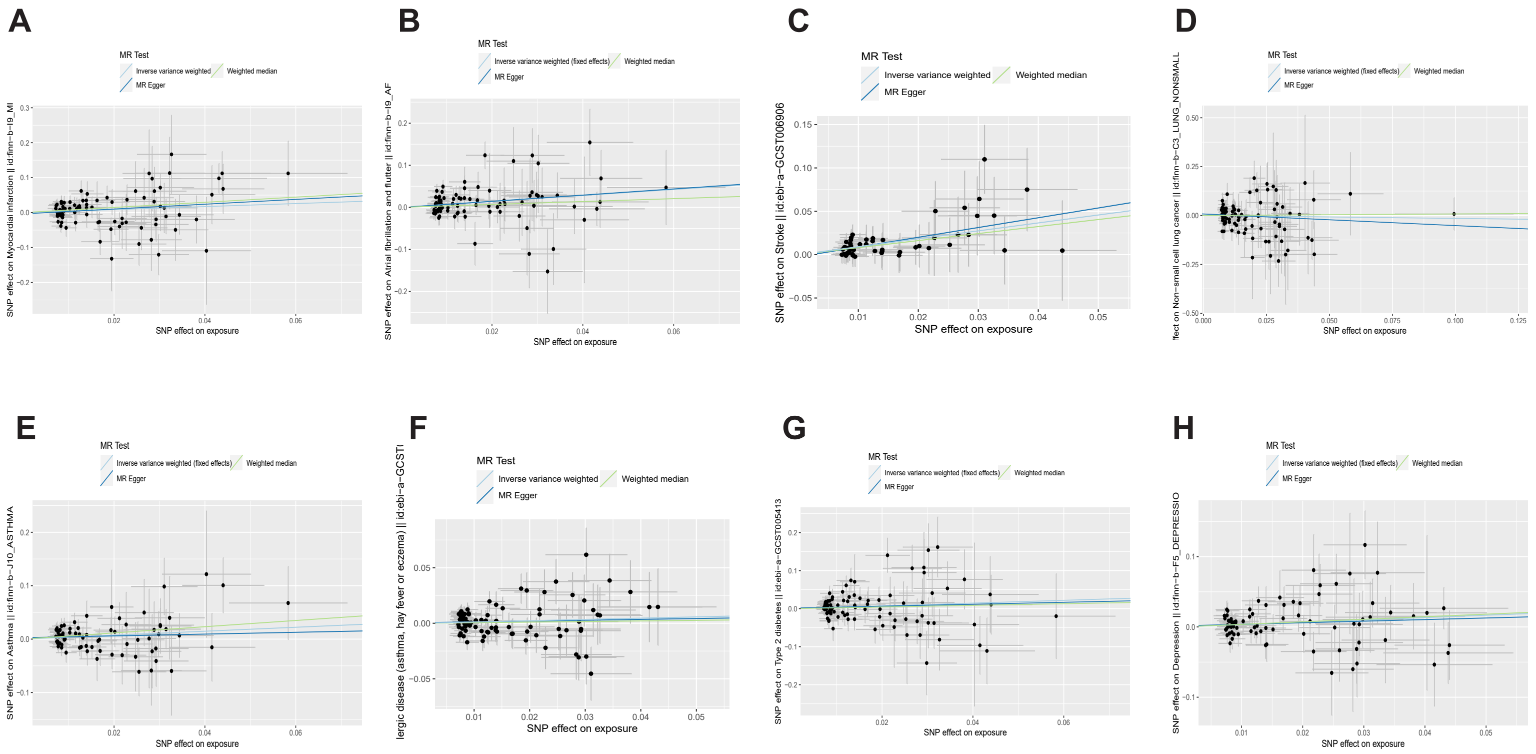

Figure S2. Scatter plot of causal effects of SHS associated SNPs on eight diseases. Figure showed SNP effect on (A) MI, (B) AF, (C) stroke, (D) non- small cell lung cancer, (E) asthma, (F) allergic disease, (G) type 2 diabetes and (H) depression.

Supplementary Figure S3

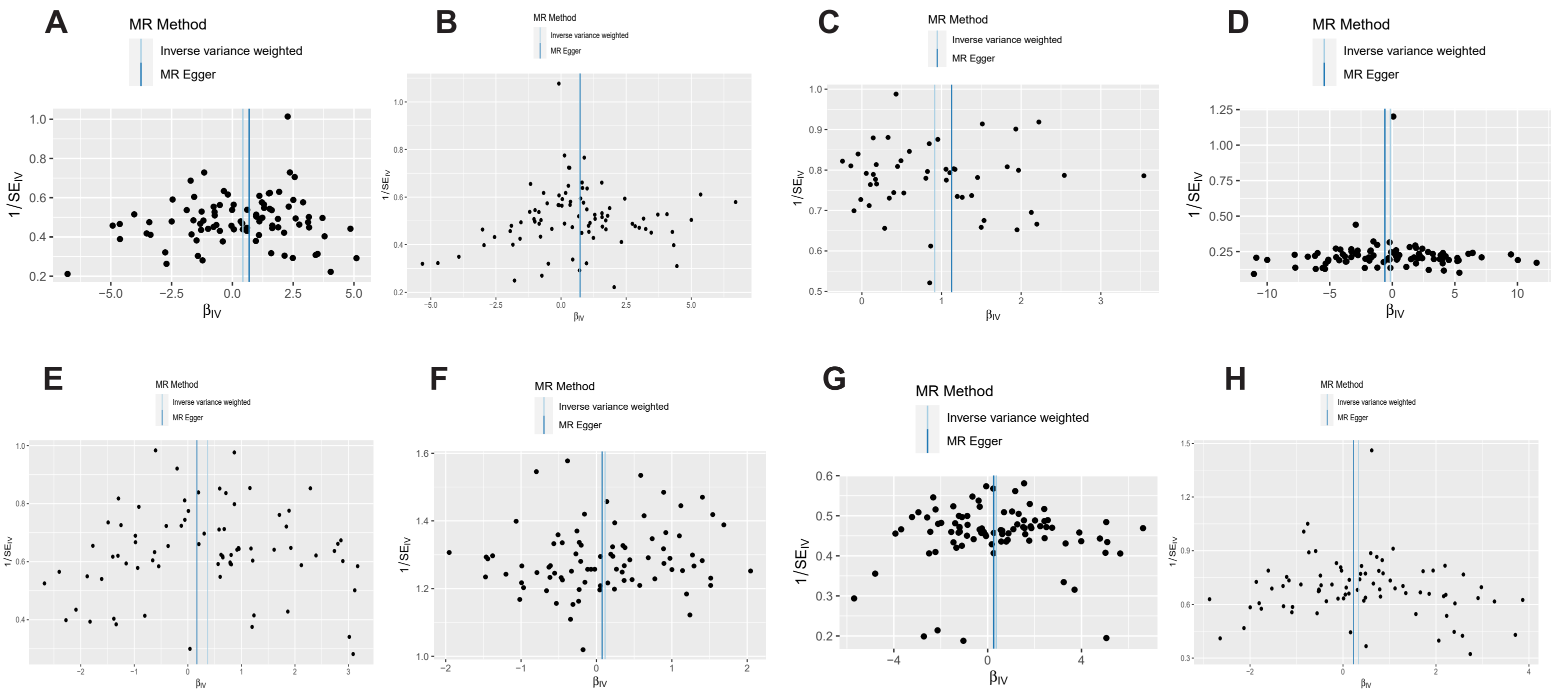

Figure S3. Funnel plot of causal effects of SHS associated SNPs on (A) MI, (B) AF, (C) stroke, (D) non-small cell lung cancer, (E) asthma, (F) allergic disease, (G) type 2 diabetes and (H) depression.

Supplementary Figure S4

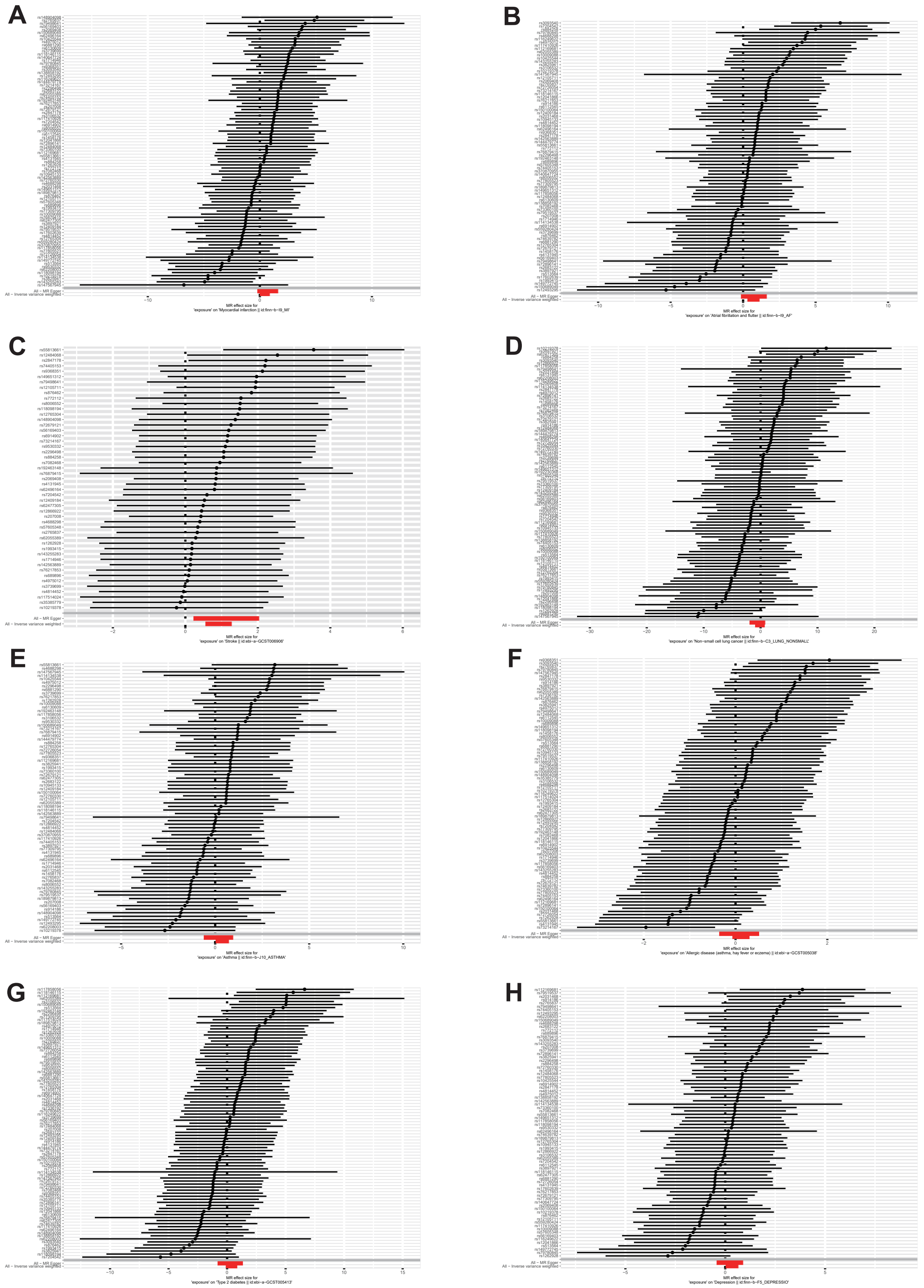

Figure S4: Forest plots for association of SHS with potential related diseases: (A) MI, (B) AF, (C) stroke, (D) non- small cell lung cancer, (E) asthma, (F) allergic disease, (G) type 2 diabetes and (H) depression.
